# Synthesis and Characterization of Impurities in the Production Process of Lopinavir

**DOI:** 10.3797/scipharm.1407-14

**Published:** 2014-09-22

**Authors:** Ambati V. Raghava Reddy, Srinivas Garaga, Chandiran Takshinamoorthy, Andra Naidu

**Affiliations:** 1 Chemical Research and Development Department, Aurobindo Pharma Ltd, Survey No: 71&72, Indrakaran Village, Sangareddy Mandal, Medak district, Hyderabad-502329, Telangana State, India; 2 Department of Chemistry, Jawaharlal Nehru Technological University Hyderabad, Kukatpally, Hyderabad, Telangana State, India; 3 Department of Engineering Chemistry, A. U. College of Engineering (A), Andhra University, Visakhapatnam-530 003, Andhra Pradesh, India

**Keywords:** Lopinavir, Related substances, Synthesis and Characterization of impurities

## Abstract

Lopinavir is an antiretroviral drug used for the inhibition of HIV protease. Four related substances of lopinavir were observed during the manufacturing process of lopinavir in the laboratory and they were identified. The present work describes the origin, synthesis, characterization, and control of these related substances.

## Introduction

Lopinavir is chemically known as (2*S*)-*N*-[(2*S*,4*S*,5*S*)-5-{[(2,6-dimethylphenoxy)acetyl]amino}-4-hydroxy-1,6-diphenylhexan-2-yl]-3-methyl-2-(2-oxotetrahydropyrimidin-1(2*H*)-yl)butanamide. Lopinavir is marketed by Abbott Laboratories under the brand name of Kaletra^®^ with the combination of Ritonavir.

The presence of impurities in an Active Pharmaceutical Ingredient (API) will influence the quality and safety of the drug product. In the regulatory guidelines of the International Conference on Harmonization (ICH), it is recommended that impurities amounting to more than 0.1% [1] should be identified and characterized. Impurities are required in pure form to check the analytical performance characteristics such as specificity, linearity, range, accuracy, precision, limit of detection (LOD), limit of quantification (LOQ), robustness, system suitability testing, and relative retention factor [2].

During the process development of lopinavir **1** in our laboratory, we observed the formation of four substances that are structurally related to lopinavir. These unknown related substances were identified, monitored, and their structures were tentatively assigned on the basis of their fragmentation patterns in LC-MS [3]. In the present work, the identified related substances of lopinavir were synthesized and characterized by various spectroscopic techniques and further confirmed by co-injection studies using qualitative HPLC analysis. A few references [3–9] were found in the literature for related substances of lopinavir and its metabolites.

## Results and Discussion

Lopinavir **1** has been synthesized by known literature methods [10–14]. Our route for the synthesis of lopinavir is shown in Scheme 1. (2*S*,3*S*,5*S*)-5-Amino-2-(dibenzylamino)-1,6-diphenylhexan-3-ol (**2**) was activated with *N,N*-carbonyldiimidazole followed by condensation with (2*S*)-3-methyl-2-(2-oxotetrahydropyrimidin-1(2*H*)-yl)butanoic acid (**3**) to provide (2*S*)-*N*-[(2*S*,4*S*,5*S*)-5-(dibenzylamino)-4-hydroxy-1,6-diphenylhexan-2-yl]-3-methyl-2-(2-oxotetrahydropyrimidin-1(2*H*)-yl)butanamide (**4**). Compound **4**, upon debenzylation with ammonium formate and palladium on charcoal, gave (2*S*)-*N*-[(2*S*,4*S*,5*S*)-5-amino-4-hydroxy-1,6-diphenylhexan-2-yl]-3-methyl-2-(2-oxotetrahydropyrimidin-1(2*H*)-yl)butanamide (**5**). Compound **5** was treated with L-pyroglutamic acid to give pure (2*S*)-*N*-[(2*S*,4*S*,5*S*)-5-amino-4-hydroxy-1,6-diphenylhexan-2-yl]-3-methyl-2-(2-oxotetrahydropyrimidin-1(2*H*)-yl)butanamide (*S*)-pyroglutamic acid salt **6**. Condensation of compound **6** with (2,6-dimethylphenoxy)acetic acid gave lopinavir **1**. Using the above-mentioned synthesis process, lopinair 1 was obtained with a purity of 99.5% and four impurities that are structurally related to lopinavir were identified. The chemical names and structures of these related substances of lopinavir **1** were identified as:

**Sch. 1.**
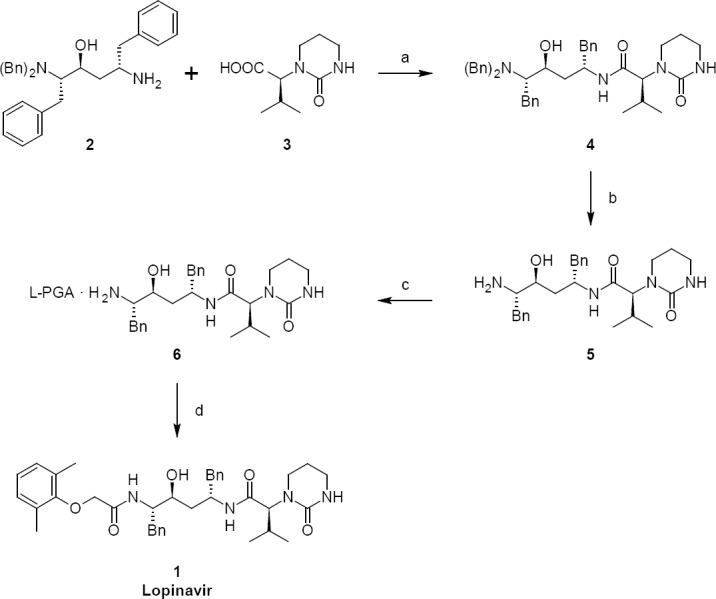
Synthesis of Lopinavir 1. Reagents and conditions: (a) *N*,*N*-carbonyldiimidazole, ethyl acetate; yield: 82.7%; (b) ammonium formate, palladium on charcoal, methanol; yield: 100%; (c) L-pyroglutamic acid, acetone, DMF; yield: 72.4%; (d) 2,6-dimethylphenoxyaceticacid, thionyl chloride, dichloromethane; yield: 85.7%

- Lopinavir dimer, **7** (2S,2’S)-N,N’-[(3,3’,5,5’-tetramethylbiphenyl-4,4’-diyl)bis{oxy(1-oxoethane-2,1-diyl)imino[(2S,4S,5S)-4-hydroxy-1,6-diphenylhexane-5,2-diyl]}]bis[3-methyl-2-(2-oxotetrahydropyrimidin-1(2H)-yl)butanamide]:
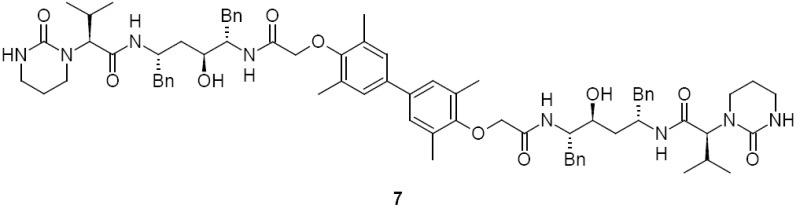
- Lopinavir carboxymethyl analog, **8** (2S)-N-[(2S,4S,5S)-4-hydroxy-5-({[4-(2-{[(2S,3S,5S)-3-hydroxy-5-{[(2S)-3-methyl-2-(2-oxotetrahydropyrimidin-1(2H)-yl)butanoyl]amino}-1,6-diphenylhexan-2-yl]amino}-2-oxoethoxy)-3,5-dimethylphenyl]acetyl}amino)-1,6-diphenylhexan-2-yl]-3-methyl-2-(2-oxotetrahydropyrimidin-1(2H)-yl)butanamide:
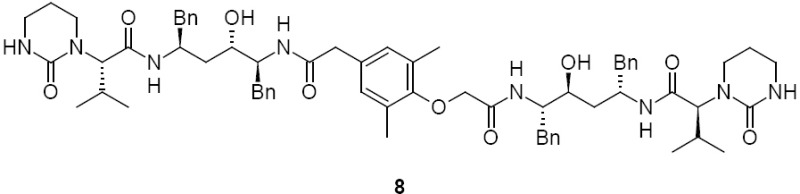
- Lopinavir diamide, **9** N,N’-[(2S,3S,5S)-3-Hydroxy-1,6-diphenylhexane-2,5-diyl]bis[2-(2,6-dimethylphenoxy)acetamide]:
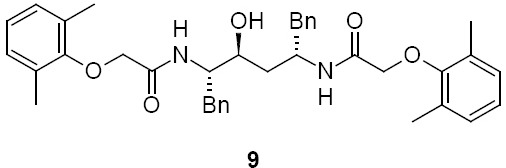
- Diacylated, **10** (2S)-N-[(2S,4S,5S)-5-{[(2,6-dimethylphenoxy)acetyl]amino}-4-hydroxy-1,6-diphenylhexan-2-yl]-2-{3-[(2,6-dimethylphenoxy)acetyl]-2-oxotetrahydropyrimidin-1(2H)-yl}-3-methylbutanamide:
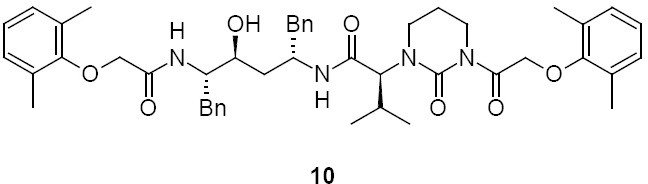

The origin, synthesis, characterization, and control of these related substances are described below. The synthetically prepared related substances were characterized by conventional spectroscopic studies and the presence of these related substances in the lopinavir batch was confirmed by spiking the related substances individually with a lopinavir sample from the manufactured batch. These studies confirmed the formation of related substances (**7–10**) during the manufacturing process of lopinavir **1**.

### Lopinavir Dimer 7

2,6-Dimethylphenol is a raw material used in the preparation of 2,6-dimethylphenoxyacetic acid (Scheme 2). 2,6-dimethylphenoxyacetic acid is a critical intermediate in the preparation of lopinavir **1** and is prepared from 2,6-dimethylphenol by acylation with chloroacetic acid by known literature methods [10–14].

**Sch. 2:**
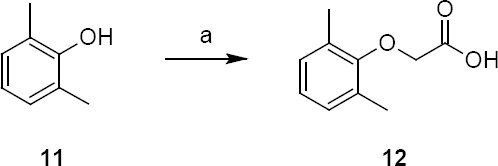
Synthetic scheme of 2,6-dimethylphenoxyacetic acid 12. Reagents and conditions: (a) chloroacetic acid, sodium hydroxide, water, reflux; yield: 90%

**Fig. 1:**
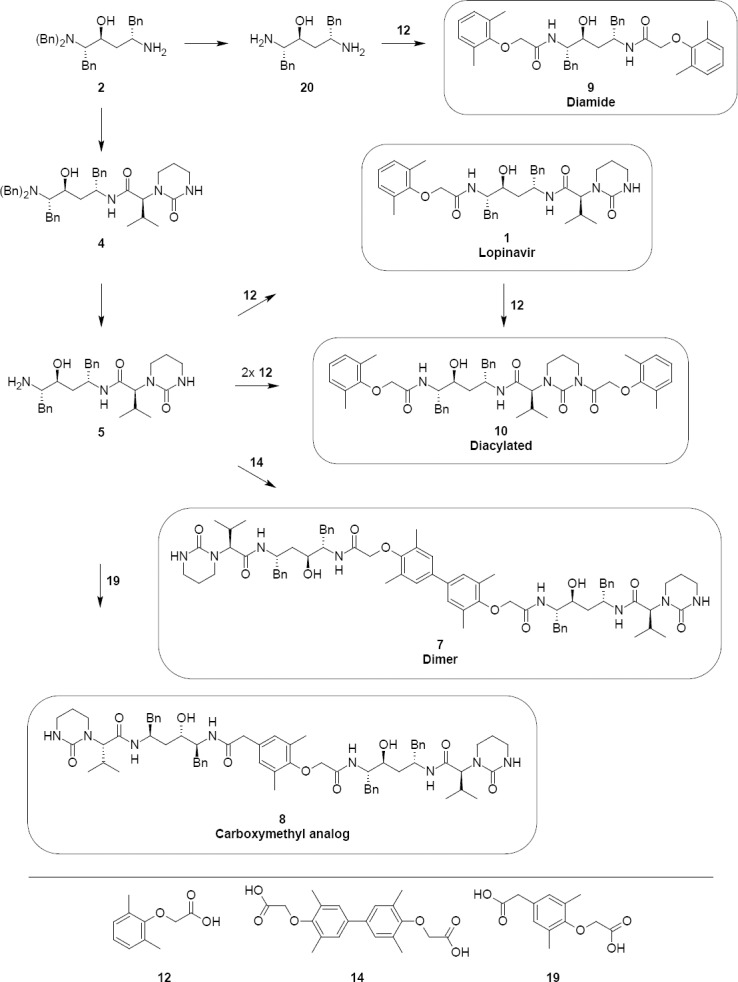
Possible synthetic pathways for lopinavir 1 and its related substances (7–10)

Phenols have a tendency to polymerize during storage; it is therefore necessary to study the dimers of phenols. We observed a dimer (**13**) at a level of ~0.3% in 2,6-dimethylphenol used in the laboratory and it was subsequently reacted to give lopinavir dimer **7**. Lopinavir dimer **7** was independently prepared by the dimerization of 2,6-dimethylphenol to give compound **13**. Compound **13** was acylated with chloroacetic acid to give compound **14**. Compound **14** was condensed with compound **4** to produce lopinavir dimer **7** (as shown in Scheme 3).

**Sch. 3:**
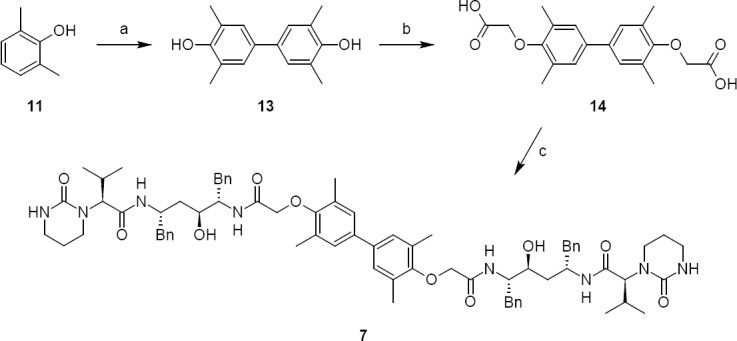
Synthetic scheme of lopinavir dimer 7. Reagents and conditions: (a) ferric chloride, water; yield: 25%; (b) chloroacetic acid, sodium hydroxide, water; yield: 50%; (c) 6, thionyl chloride, dichloromethane, sodium bicarbonate, water, ethyl acetate; yield: 40%

### Lopinavir Carboxymethyl Analog 8

During the acylation of 2,6-dimethylphenol with chloroacetic acid (as shown in Scheme 2), the formation of ~0.2% of compound **19** was observed and was reacted in the subsequent steps to give lopinavir carboxymethyl analog **8**.

Lopinavir carboxymethyl analog **8** was independently prepared from 2,6-dimethylphenol. 2,6-dimethylphenol reacted with acetic anhydride to give 2,6-dimethylphenyl acetate **15**. 2,6-dimethylphenyl acetate **15** underwent fries rearrangement which gave para acetyl 2,6-dimethylphenol **16**. Compound **16** reacted with chloroacetic acid to give compound **17**. Compound **17** was treated with morpholin to give compound **18**. Compound **18** was hydrolyzed with sodium hydroxide to give compound **19**. Compound **19** condensed with compound **5** to give lopinavir carboxymethyl analog **8** (as shown in Scheme 4).

**Sch. 4:**
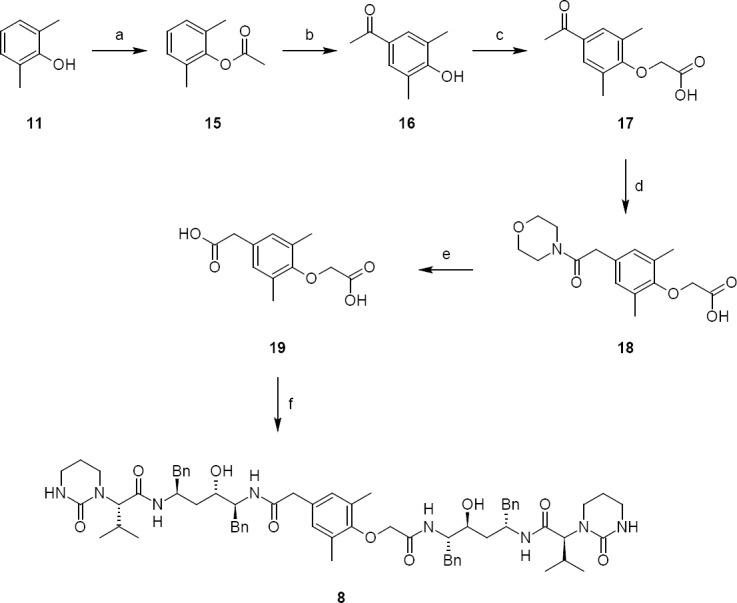
Synthetic scheme of lopinavir carboxymethyl analog 8. Reagents and conditions: (a) acetic anhydride, sulfuric acid; yield: 77%; (b) aluminium chloride, carbon disulfide, 120°C; yield: 81%; (c) chloroacetic acid, NaOH, water; yield: 70%; (d) morpholin, sulphur, NaOH; yield; 48%; (f) 5, thionyl chloride, dichloromethane, NaHCO_3_, water; yield; 38%

### Lopinavir Diamide Impurity 9

Lopinavir diamide **9** originated from unreacted compound **2**, present also in compound **4**, underwent all the reaction sequences employed in the synthesis of lopinavir. Lopinavir diamide impurity **9** was formed by the condensation of diamine compound **20** with 2,6-dimethylphenoxyacetic acid **12**.

**Sch. 5:**
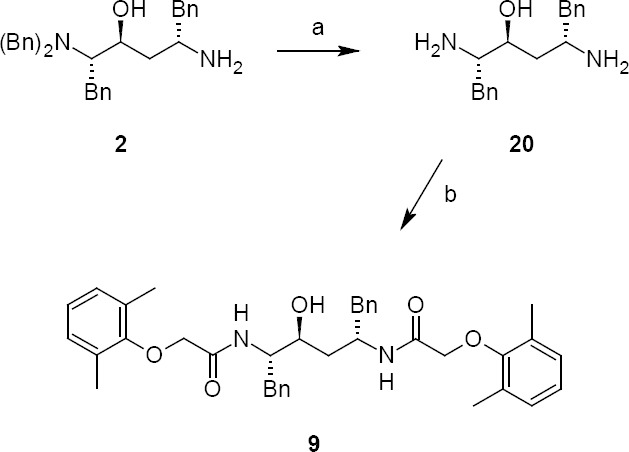
Synthetic scheme of lopinavir diamide impurity 9. Reagents and conditions: (a) ammonium formate, palladium on charcoal, methanol; yield: 100%; (b) (2,6-dimethylphenoxy)acetic acid, sodium bicarbonate; yield: 41%

### Lopinavir Diacylated Impurity 10

Lopinavir diacylated impurity **10** was formed due to the acylation on the nitrogen presented in the pyrimidine ring of lopinavir with 2,6-dimethylphenoxy acetic acid during the synthesis of lopinavir. It was independently prepared by the condensation of compound **1** with compound **12**.

**Sch. 6:**
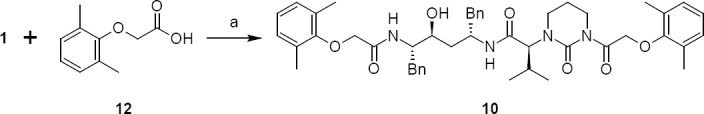
Synthetic scheme of lopinavir diacylated impurity 10. Reagents and conditions: (a) thionyl chloride, sodium bicarbonate; ethyl acetate; yield: 20%

Related substances **8–10** were eliminated during the purification of lopinavir **1**. Related substance **7** originated due to the presence of dimer **13** in 2,6-dimethylphenol. It was controlled by keeping the limit in the specification of 2,6-dimethylphenol raw material.

## Experimental

All melting points were determined with the Polmon melting point apparatus. ^1^H-NMR and ^13^C-NMR spectra were recorded on a Bruker 300 spectrometer. Chemical shifts are reported in ppm downfield from TMS as the internal standard. The mass spectra were measured on the Perkin Elmer PE SCIEX-API 2000 mass spectrometer. Elemental analyses were performed using a Heraeus CHN-O-Rapid instrument. Analytical HPLC were run with the Symmetry C_18_, 210 × 4.6 mm column at 290 nm. “RT” denotes room temperature. 2,6-dimethylphenol was purchased from Bluestar Newchemicals Materials Co., Ltd.

### (2S,2’S)-N,N’-[(3,3’,5,5’-tetramethylbiphenyl-4,4’-diyl)bis{oxy(1-oxoethane-2,1-diyl)imino[(2S,4S,5S)-4-hydroxy-1,6-diphenylhexane-5,2-diyl]}]bis[3-methyl-2-(2-oxotetrahydropyrimidin-1(2H)-yl)butanamide] (lopinavir dimer, 7):

#### Step I: 3,3^1^,5,5^1^-Tetramethyl-1,1^1^-biphenyl-4,4^1^-diol (**13**):

To a solution of 2,6-dimethylphenol 11 (10 g, 82 mmol) in DM water (400 mL) was added ferric chloride solution (22.15 g of ferric chloride·6 H_2_O dissolved in 100 mL DM water) in 30 min at 95–100°C. After stirring for 1 h at 95–100°C, the reaction mass cooled to room temperature and we extracted the product with dichloromethane (250 mL). The dichloromethane layer was concentrated below 40°C under reduced pressure to obtain a brown coloured residue, which was purified by silicagel (100–200 mesh) chromatography using a 1:9 v/v mixture of ethylacetate and hexane to obtain 5 g (25%) of 3,3^1^,5,5^1^-tetramethyl-1,1^1^-biphenyl-4,4^1^-diol (13) as an orange colored residue. IR (KBr, cm^−1^) 2883, 2855, 1633, 1595, 1462, 1364, 1343, 1296, 780, 766; ^1^H-NMR (300 MHz, DMSO-D_6_) δ 2.19 (S, 12H), 3.35 (S, 3H), 7.1 (S, 4H), 8.13 (S, 2H); MS (ESI, *m/z*): 237 [M-H]^-^.

#### Step II: 2,2^1^-[(3,3^1^,5,5^1^-tetramethylbiphenyl-4,4^1^-diyl)bis(oxy)]diacetic acid (**14**):

To a suspension of 3,3^1^,5,5^1^-tetramethyl-1,1^1^-biphenyl-4,4^1^-diol (**13**, 7.0 g, 28.92 mmol) and chloroacetic acid (5.46 g, 57.77 mmol) in DM water (50 mL) was added 10% aqueous sodium hydroxide (50 mL) in 10 min at 25–35°C. The reaction mass was heated and refluxed for ~2 h at 98-100°C. The reaction mass cooled to 25-30°C and we adjusted the pH of the reaction mass to 5.0 with concentrated hydrochloric acid (~6 mL) and it was then washed with toluene (50 mL). Thereafter, we precipitated the product by adjusting the pH to 2.0 with concentrated hydrochloric acid (~6 mL) at 25-30°C. After 1 h of stirring at 25-30°C, the product was filtered and dried to give 7.3 g (70%) of crude 2,2^1^-[(3,3^1^,5,5^1^-tetramethyl biphenyl-4,4^1^-diyl)bis(oxy)]diacetic acid **14**, which was used as such in the next step without purification.

#### Step III: (2S,2’S)-N,N’-[(3,3’,5,5’-tetramethylbiphenyl-4,4’-diyl)bis{oxy(1-oxoethane-2,1-diyl)imino[(2S,4S,5S)-4-hydroxy-1,6-diphenylhexane-5,2-diyl]}]bis[3-methyl-2-(2-oxotetrahydropyrimidin-1(2H)-yl)butanamide] (lopinavir dimer, **7**):

To a suspension of 2,2^1^-[(3,3^1^,5,5^1^-tetramethylbiphenyl-4,4^1^-diyl)bis(oxy)]diacetic acid (**14**, 4.0 g, 11.66 mmol) in ethyl acetate (20 mL) was added thionyl chloride (3.32 g, 27.89 mmol) followed by *N*,*N*-dimethylformamide (0.1 mL) at 25–30°C. The reaction mass temperature was raised and it was stirred for 2 h at 50-55°C to complete the formation of acid chloride. Compound 6 (13.3 g, 22.35 mmol) was dissolved separately in a mixture of water (100 mL) and ethyl acetate (100 mL) at 20–25°C, then NaHCO_3_ was added (11.42 g, 135.95 mmol) portionwise at 20–25°C. Thereafter, we added the above-prepared acid chloride solution in ~15 min at 20–25°C. After 30 min of stirring at 20–25°C, we separated the aqueous layer from the reaction mass and washed the organic layer with 5% aqueous sodium bicarbonate (50 mL) followed by 10% aqueous sodium chloride (50 mL). The organic layer was concentrated at 50–60°C under reduced pressure to obtain 20 g of cream coloured powder, which was purified by flash chromatography using a 1:1 v/v mixture of hexane and ethyl acetate to obtain 14 g (40%) of pure lopinavir dimer (**7**). Melting range: 145–149°C; HPLC purity: 90.11%; IR (KBr, cm^−1^): 3839, 3746, 3407, 3318, 3061, 3028, 2961, 2927, 2870, 1651, 1516, 1452, 1348, 1308, 1195, 750, 701; ^1^H-NMR (300 MHz, DMSO-D_6_) δ 0.72 (d, 12H), 1.15 & 1.19 (2m, 8H), 1.96 & 2.11 (2*m*, 2H), 2.06 (s, 6H), 2.44 & 2.80 (2m, 10H), 2.9 (Abq, 4H), 3.02 (Abq, 2H), 3.4&3.5 (2d, 2H), 4.30 (d, 2H), 4.82 & 4.99 (2d, 2H), 6.30 (brs, 2H), 6.78 (S, 2H), 7.43 (d,2H), 7.57 & 7.71 (2d, 2H); ^13^C-NMR (75 MHz, DMSO-D_6_) δ: 16.1, 18.7, 19.6, 21.7, 25.5, 37.9, 38.7, 39.2, 39.5, 39.8, 40.1, 46.7, 52.6, 61.4, 68.4, 70.3, 125.6, 125.9, 126.9, 127.9, 129.1, 130.6, 135.6, 138.9, 139.1, 153.9, 155.4, 167.3, 169.3. HRMS: 1255.7251.

### (2S)-N-[(2S,4S,5S)-4-hydroxy-5-({[4-(2-{[(2S,3S,5S)-3-hydroxy-5-{[(2S)-3-methyl-2-(2-oxotetrahydropyrimidin-1(2H)-yl)butanoyl]amino}-1,6-diphenylhexan-2-yl]amino}-2-oxoethoxy)-3,5-dimethylphenyl]acetyl}amino)-1,6-diphenylhexan-2-yl]-3-methyl-2-(2-oxotetrahydropyrimidin-1(2H)-yl)butanamide (lopinavir carboxymethyl analog, 8):

#### Step I: 2,6-dimethylphenyl acetate (**15**):

To a solution of acetic anhydride (167 g, 1637 mmol) and concentrated sulfuric acid (5 g, 51 mmol) was added 2,6-dimethylphenol (**11**, 100 g, 819.6 mmol) in 30 min at 25–30°C. After stirring at 25–30°C for 1 h, we poured the reaction mass into cold water (500 mL) and extracted the product with dichloromethane. The organic layer was washed with 5% w/w aqueous sodium bicarbonate (500 mL) followed by water (500 mL). The organic layer was concentrated below 40°C under reduced pressure to obtain 103 g (77%) of 2,6-dimethylphenyl acetate (**15**) as an oily mass. IR (KBr, cm^−1^) 2950, 1590, 1494, 1439, 1381, 1350, 788, 764**;** ^1^H-NMR (300 MHz, CDCl_3_) δ: 2.15 (s, 3H), 2.26 (s, 6H), 7.05 (s, 3H); MS (ESI, *m/z*): 165 [M+H]^+^.

#### Step II: 1-(4-hydroxy-3,5-dimethylphenyl)ethanone (**16**):

To a suspension of aluminum chloride (105.6 g, 792.19 mmol) in carbon disulfide (200 mL) was added 2,6-dimethylphenyl acetate (**15**, 100 g, 609.75 mmol) in 20 min at 25–30°C. After 2 h of stirring at 39–40°C, carbon disulfide was distilled out and we raised the mass temperature to 120–125°C. After 6 h of stirring at 120-125°C, we cooled the mass to 20–25°C and added concentrated hydrochloric acid (50 mL) followed by water (500 mL) and stirred for 1 h at 30–50°C. Thereafter, the mass was cooled to 20–25°C and the product was extracted with dichloromethane (2 × 500 mL). The organic layer was concentrated below 40°C under reduced pressure to obtain a residue, further this residue crystallized from ethyl acetate (200 mL) to get 81 g (81%) of pure 1-(4-hydroxy-3,5-dimethylphenyl)ethanone (**16**). IR (KBr, cm^−1^) 2950, 1590, 1494, 1439, 1381, 1350, 788, 764; ^1^H-NMR (300 MHz, CDCl_3_) δ 2.22 (s, 6H), 2.39 (s, 3H), 7.58 (s, 2H), 9.15 (1H, OH); MS (ESI, *m/z*): 165 [M+H]^+^.

#### Step III: (4-Acetyl-2,6-dimethylphenoxy)acetic acid (**17**):

To a suspension of 1-(4-hydroxy-3,5-dimethylphenyl)ethanone (**16**, 60 g, 365.85 mmol) and chloroacetic acid (37.12 g, 392 mmol) in DM water (300 mL) was added 20% w/w aqueous sodium hydroxide (150 g, 750 mmol) in 10 min at 25–30°C. After stirring for 1 h at reflux temperature (98–100°C), the mass was cooled to 20–25°C and we adjusted the pH of the reaction mass to 5.0 with concentrated hydrochloric acid (40 mL, 430 mmol). The reaction mass was washed with toluene (200 mL) to remove the unreacted starting material and then we adjusted the aqueous layer pH to 2.0 with concentrated hydrochloric acid (35 mL, 370 mmol) at 20–25°C. The precipitated product was collected by filtration and dried at 45–50°C under reduced pressure to obtain 57 g (70%) of pure [4-acetyl-2,6-dimethylphenoxy)acetic acid (**17**). IR (KBr, cm^−1^) 2883, 2855, 1633, 1595, 1462, 1364, 1343, 1296, 780, 766; ^1^H-NMR (300 MHz, DMSO-D_6_) δ 2.29 (s, 6H), 2.46 (s, 3H), 4.45 (s, 2H), 7.65 (s, 2H); MS (ESI, *m/z*): 221 [M-H]^-^.

#### Step IV: [4-(Carboxymethoxy)-3,5-dimethylphenyl]acetic acid (**19**):

A mixture of (4-acetyl-2,6-dimethylphenoxy)acetic acid (**17**, 25 g, 112 mmol) and sulfur powder (5.4 g, 168 mmol) in morpholine (24.52 g, 281 mmol) was heated to 130°C and stirred for 12 h at 130°C. The reaction mass was cooled to 20–25°C and we added 10% w/w ethanolic sodium hydroxide solution (150 g, 375 mmol). Again, the mass temperature was raised to 85 °C and stirred for 10 h at 85°C. Thereafter, the reaction mass was concentrated at 85°C under reduced pressure, the obtained residue was dissolved in DM water (250 mL) and washed with ethyl acetate (250 mL). The aqueous layer was adjusted to pH 5.0 with concentrated hydrochloric acid (24 mL) at 20–25°C and again washed with ethyl acetate (125 mL) at 20–25°C. After that, the aqueous layer was adjusted to pH 1.0 with concentrated hydrochloric acid (12 mL) at 20–25°C and we extracted the product with dichloromethane (4X 100 mL). The combined dichloromethane layer was concentrated below 40°C under reduced pressure to obtain a gummy solid, which was crystallized from ethyl acetate and hexane to obtain 15 g (46%) of pure [4-(carboxymethoxy)-3,5-dimethylphenyl]acetic acid (**19**) as a pale yellow solid. IR (KBr, cm^−1^) 2883, 2855, 1633, 1595, 1462, 1364, 1343, 1296, 780, 766; ^1^H-NMR (300 MHz, DMSO-D_6_) δ 2.20 (s, 6H), 3.35 (s, 3H), 4.34 (s, 2H), 6.89 (s, 2H), 12.58 (brs 2H); MS (ESI, *m/z*): 237 [M-H]^-^.

#### Step V: (2S)-N-[(2S,4S,5S)-4-hydroxy-5-({[4-(2-{[(2S,3S,5S)-3-hydroxy-5-{[(2S)-3-methyl-2-(2-oxotetrahydropyrimidin-1(2H)-yl)butanoyl]amino}-1,6-diphenylhexan-2-yl]amino}-2-oxoethoxy)-3,5-dimethylphenyl]acetyl}amino)-1,6-diphenylhexan-2-yl]-3-methyl-2-(2-oxotetrahydropyrimidin-1(2H)-yl)butanamide (lopinavir carboxymethyl analog, **8**):

To a suspension of [4-(carboxymethoxy)-3,5-dimethylphenyl]acetic acid (**18**, 10 g, 42 mmol) in ethyl acetate (50 mL) was added thionyl chloride (11.0 g, 92.43 mmol) followed by *N*,*N*-dimethylformamide (0.1 mL) at RT. The reaction mass was heated to 45–50°C and stirred for 2 h at 45–50°C to complete the formation of acid chloride. Compound **6** (50 g, 84 mmol) was dissolved separately in a mixture of DM water (200 mL) and ethyl acetate (200 mL) at 20–25°C, then NaHCO_3_ (26.8 g, 319 mmol) was added portionwise at 20–25°C. Thereafter, we added the above-prepared acid chloride solution in ~30 min at 20–25°C. After 30 min of stirring at 20–25°C, the aqueous layer was separated from the reaction mass and the organic layer was washed with 3 N hydrochloric acid (200 mL) followed by DM water (200 mL). Finally, the organic layer was washed with 10% aqueous sodium chloride (200 mL). The organic layer was concentrated at 50–60°C under reduced pressure to obtain a cream coloured powder, which was purified by flash chromatography using a 1:1 v/v mixture of hexane and ethyl acetate to obtain 18 g (38%) of pure compound **8**. Melting range: 140–145 °C; HPLC purity: 89.22%; IR (KBr, cm^−1^): 3856, 3410, 3326, 3062, 3028, 2963, 2931, 2871, 1644, 1514, 1452, 1307, 1209, 1180, 749, 701; ^1^H-NMR (300 MHz, DMSO-D_6_) δ 0.74 (d, 12H), 1.43 & 1.51 (2m, 8H), 1.96 & 2.11 (2*m*, 2H), 2.06 (s, 6H), 2.44-2.81(m, 8H), 2.91 & 3.01 (2m, 8H), 3.25 (m, 2H), 3.54 & 3.64 (2m, 2H), 3.93 & 4.00 (Abq, 2H), 4.17 & 4.25 (2m, 4H), 4.30 (d, 2H), 4.82 & 4.99 (2d, 2H), 6.30 (brs, 2H), 6.78 (s, 2H), 7.04-7.23 (m, 2H, Ar), 7.43 (d, 2H), 7.57 & 7.71 (2d, 2H); ^13^C-NMR (75 MHz, DMSO-D_6_) δ: 14.9, 16.7, 19.5, 20.4, 21.6, 22.5, 23.7, 26.3, 38.4, 38.8, 39.5, 39.8, 40.1, 40.4, 40.9, 41.2, 42.5, 47.6, 53.4, 53.7, 60.6, 62.3, 68.2, 69.3, 70.0, 71.1, 126.3, 126.8, 128.6, 129.9, 130.1, 130.8, 133.4, 139.7, 140.4, 153.7, 156.3, 168.1, 170.0, 170.2, 170.9. HRMS: 1135.6671.

### N,N’-[(2S,3S,5S)-3-Hydroxy-1,6-diphenylhexane-2,5-diyl]bis[2-(2,6-dimethylphenoxy)acetamide] (lopinavir diamide, 9):

#### Step I: (2*S*,3*S*,5*S*)-2,5-diamino-1,6-diphenylhexan-3-ol (**20**):

To a solution of (2*S*,3*S*,5*S*)-5-amino-2-(dibenzylamino)-1,6-diphenylhexan-3-ol (**2**, 50 g, 108 mmol) in methanol was added ammonium formate (20.41 g, 320 mmol) followed by 5% palladium on charcoal (5 g, 50% wet) at room temperature. After raising the temperature to 50°C, the reaction was stirred for ~2 h at 50°C to complete the debenzylation. Thereafter, the reaction mass cooled to room temperature and we recovered the palladium on charcoal by filtration under a nitrogen atmosphere. The methanol filtrate was concentrated at 60°C under reduced pressure which resulted in a residue. This residue was dissolved in ethyl acetate (250 mL) and washed with water (100 mL), followed by 20% aqueous sodium chloride (100 mL). The ethyl acetate layer was concentrated at 60°C under reduced pressure to obtain 30 g (100%) of title compound **20** as a foamy solid.

#### Step II: N,N’-[(2S,3S,5S)-3-Hydroxy-1,6-diphenylhexane-2,5-diyl]bis[2-(2,6-dimethylphenoxy)acetamide] (lopinavir diamide, **9**):

To a suspension of (2,6-dimethylphenoxy)acetic acid (**12**, 38 g, 211 mmol) in ethyl acetate (100 mL) was added thionyl chloride (31.40 g, 264 mmol) followed by *N*,*N*-dimethylformamide (0.1 mL) at 25–30°C. The reaction mass temperature was raised to 50–55°C and stirred for 2 h at 50–55°C to complete the formation of the acid chloride. We separately dissolved (2*S*,3*S*,5*S*)-2,5-diamino-1,6-diphenylhexan-3-ol (**20**, 30 g, 105 mmol) in a mixture of DM water (250 mL) and ethyl acetate (250 mL) at 20-25°C, then added NaHCO_3_ (55.4 g, 660 mmol) portionwise at 20–25°C. Thereafter, the above-prepared acid chloride solution was added in ~15 min at 20–25°C. After 30 min of stirring at 20–25°C, the aqueous layer was separated from the reaction mass and the organic layer was washed with 5% aqueous sodium bicarbonate solution (250 mL) followed by 10% aqueous sodium chloride solution (250 mL). The organic layer was concentrated at 50–60°C under reduced pressure to obtain 62.0 g of cream coloured powder, which was purified by flash chromatography using a 1:1 v/v mixture of hexane and ethyl acetate to obtain 27 g (41%) of title compound **9**. Melting range: 158–162°C; HPLC purity: 95.01%; IR (KBr, cm^−1^): 3403, 3302, 3060, 3029, 2923, 2856, 1652, 1529, 1454, 1351, 1306, 1196, 740, 698; ^1^H-NMR (300 MHz, DMSO-D_6_) δ 1.65 (m, 2H), 2.14 & 2.16 (2s, 12H), 2.78 & 2.85 (2*d*, 4H), 3.70 (m, 1H), 3.95 & 4.05 (ABq, 2H), 4.11 (s, 2H), 4.35 (m, 2H), 5.08 (d, 1H), 6.93-7.27 (m, 16H, Ar), 7.52 & 7.86 (2d, 2H); HRMS:609.3342.

### (2S)-N-[(2S,4S,5S)-5-{[(2,6-dimethylphenoxy)acetyl]amino}-4-hydroxy-1,6-diphenylhexan-2-yl]-2-{3-[(2,6-dimethylphenoxy)acetyl]-2-oxotetrahydropyrimidin-1(2H)-yl}-3-methylbutanamide (diacylated, 10):

To a suspension of (2,6-dimethylphenoxy)acetic acid (10 g, 55.55 mmol) in ethyl acetate (40 mL) was added thionyl chloride (7.93 g, 66.66 mmol) followed by *N*,*N*-dimethylformamide (0.1 mL) at 25–30°C. The reaction mass temperature was raised to 50–55°C and stirred for 2 h at 50–55°C to complete the formation of acid chloride. Compound **1** (34.88 g, 55.55 mmol) was dissolved separately in a mixture of water (100 mL) and ethyl acetate (100 mL) at 20–25°C, then we added sodium bicarbonate (27.20 g, 323.88 mmol) at 20–25°C. Thereafter, the above-prepared acid chloride solution was added in 10 min at 20–25°C. After 30 min of stirring, we separated the organic layer and washed it with 5% aqueous sodium bicarbonate (50 mL) followed by 10% aqueous sodium chloride (50 mL). The organic layer was concentrated at 55–60°C under reduced pressure to obtain 10 g of white coloured powder, which was purified by chromatography using a 1:1 v/v mixture of ethyl acetate and hexanes to obtain 2 g (20%) of compound **10**. Melting range: 79-84 °C; HPLC purity: 88.26%; IR (KBr, cm^−1^): 3856, 3748, 3410, 3062, 3028, 2963, 2925, 2873, 1662, 1529, 1454, 1358, 1195, 750, 701; ^1^H-NMR (300 MHz, DMSO-D_6_) δ 0.74 (2d, 6H), 1.45-1.71 (2m, 4H), 2.02 (m, 1H), 2.15 & 2.24 (2s, 12H), 2.47, 2.97 & 3.53 (3m, 4H), 2.71 & 2.79 (2m, 4H), 3.62 (m, 1H), 4.05 & 4.15 (Abq, 2H), 4.24 (d, 1H), 4.28 & 4.37 (2m, 2H), 4.87 & 4.96 (ABq, 2H), 5.01 (d, 1H), 6.93-7.26 (m, 16H), 7.49-7.73 (2d, 2H, Ar); HRMS: 791.4399.

**Fig. 1S:**
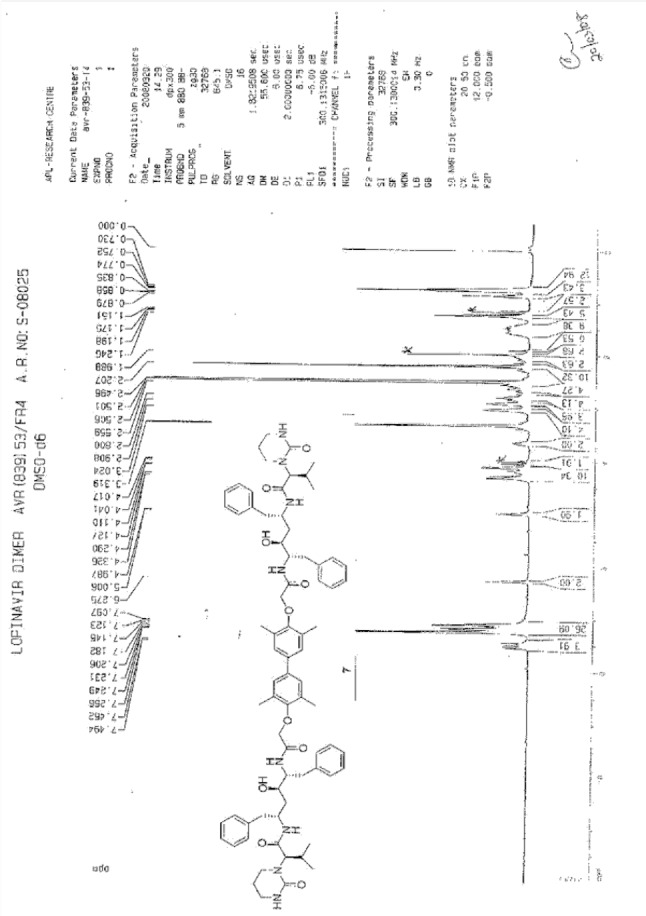
^1^H-NMR spectrum of Lopinavir dimer **7**

**Fig. 2S:**
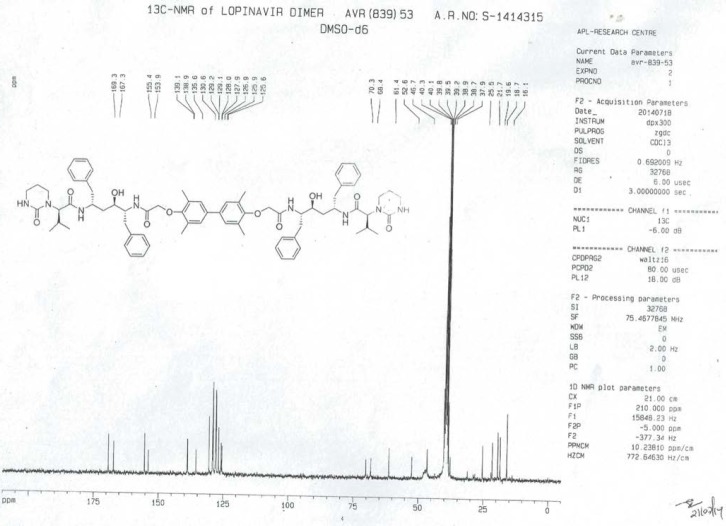
^13^C-NMR spectrum of Lopinavir dimer **7**

**Fig. 3S:**
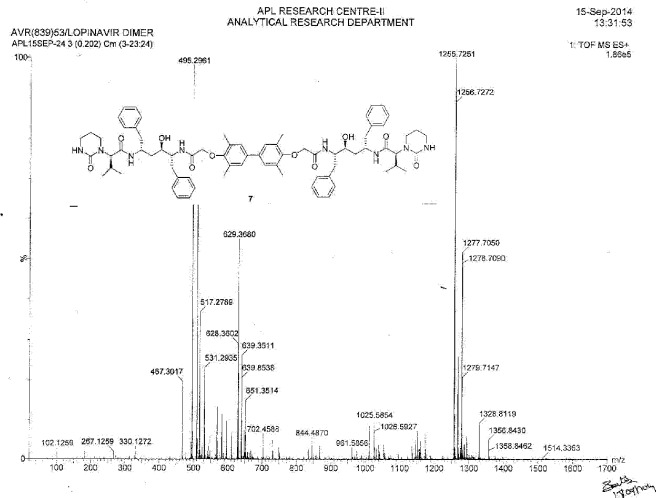
MS spectrum of Lopinavir dimer **7**

**Fig. 4S:**
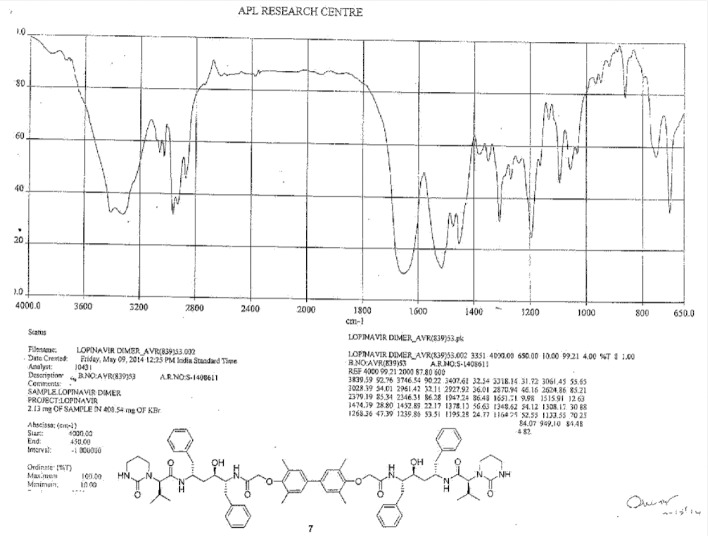
IR spectrum of Lopinavir dimer **7**

**Fig. 5S:**
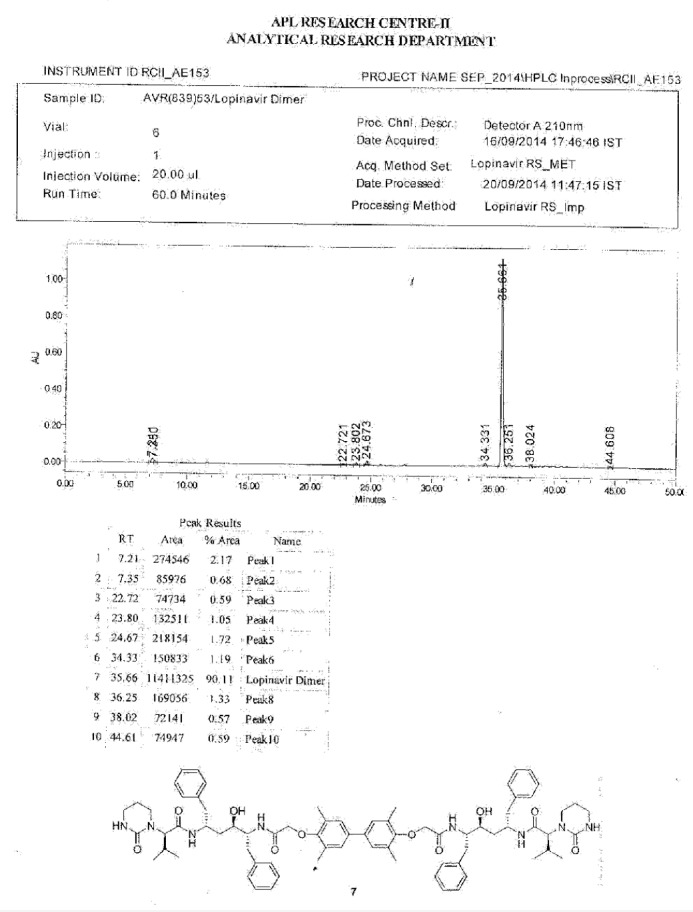
HPLC of Lopinavir dimer **7**

**Fig. 6S:**
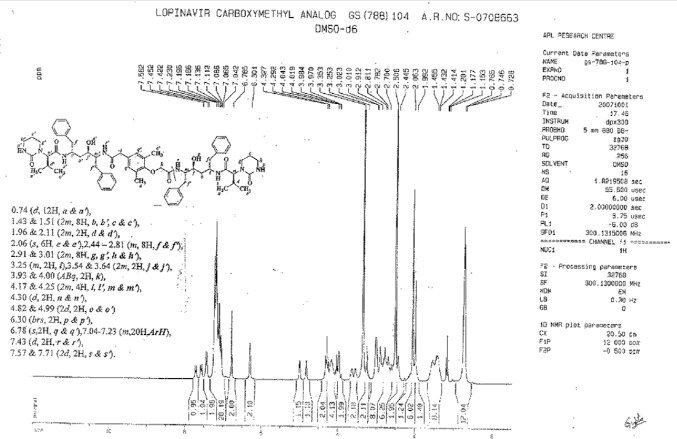
^1^H-NMR spectrum of Lopinavir carboxymethyl analog 8

**Fig. 7S:**
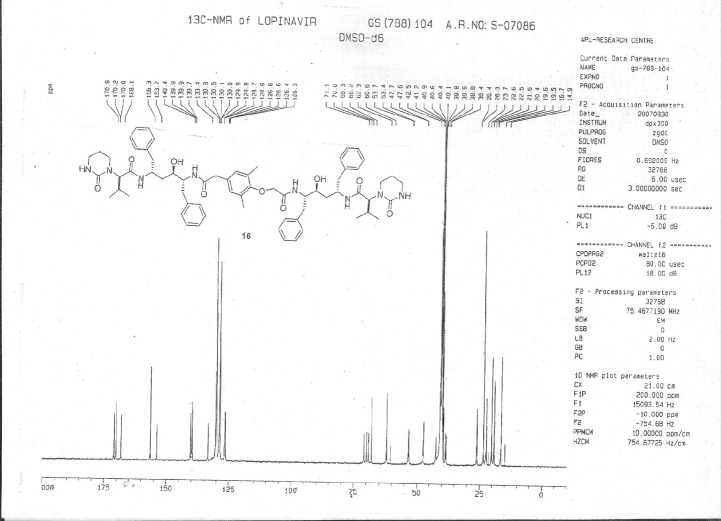
^13^C-NMR spectrum of Lopinavir carboxymethyl analog **8**

**Fig. 8S:**
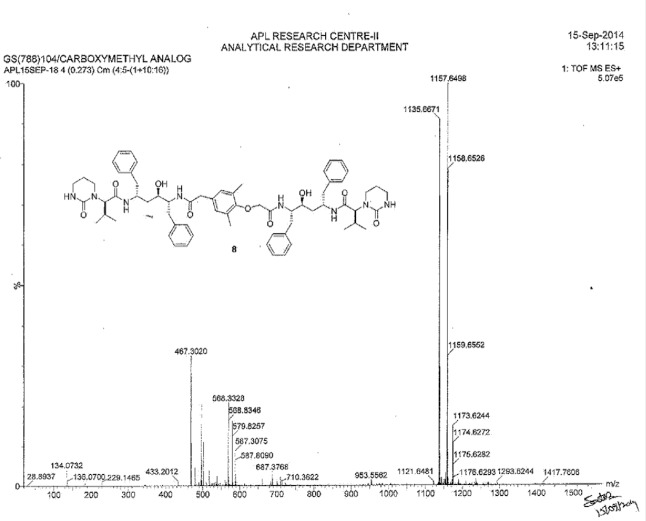
MS spectrum of Lopinavir carboxymethyl analog **8**

**Fig. 9S:**
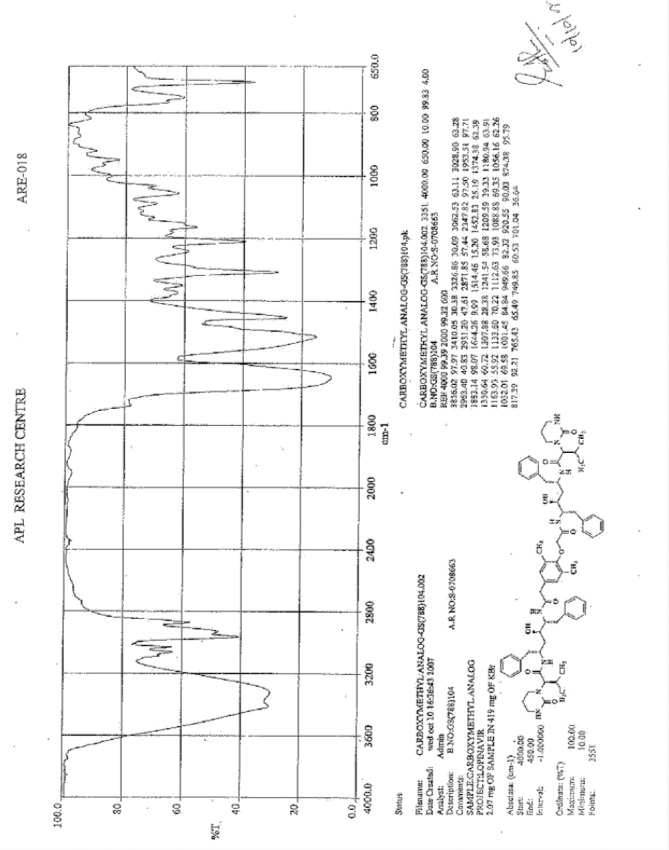
IR spectrum of Lopinavir carboxymethyl analog **8**

**Fig. 10S:**
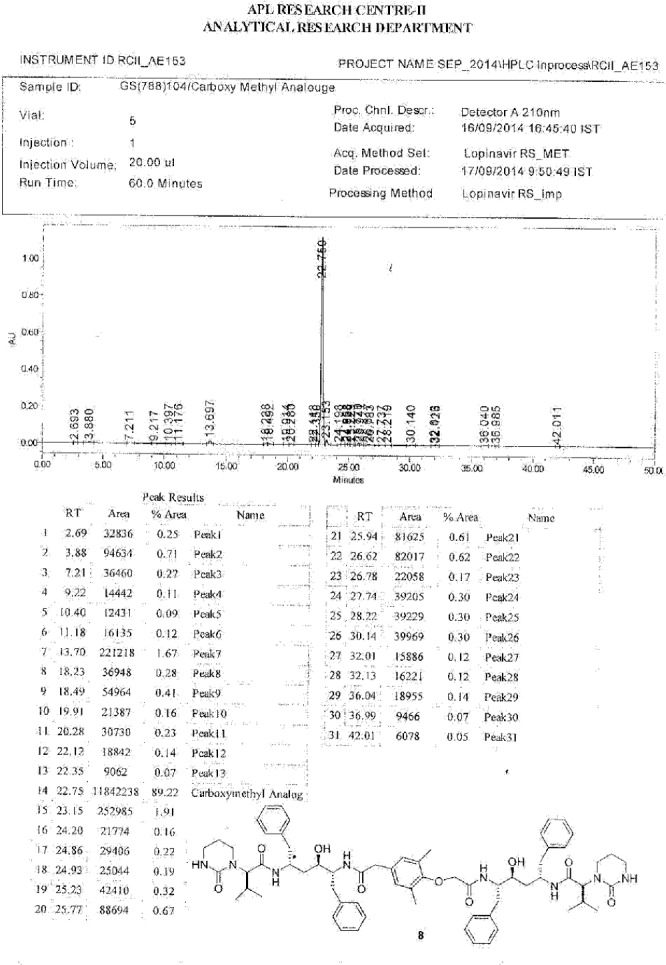
HPLC of Lopinavir carboxymethyl analog **8**

**Fig. 11S:**
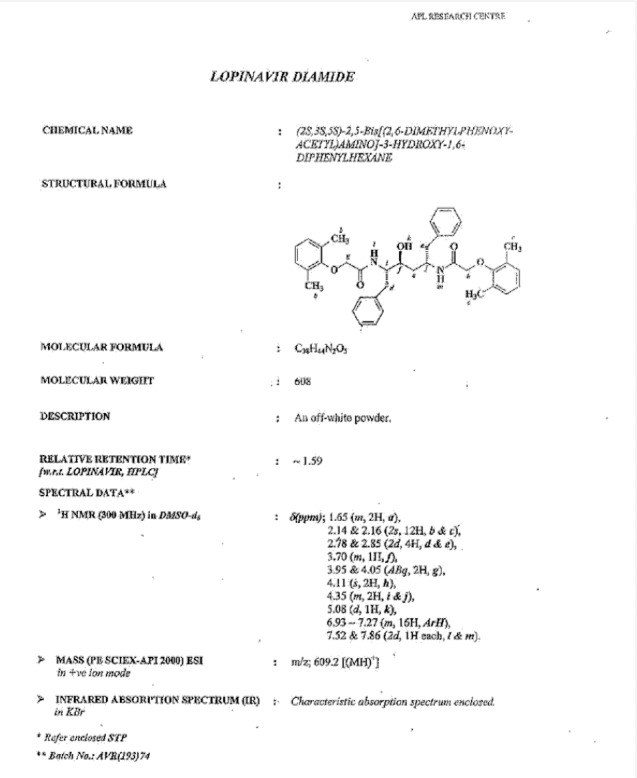
Data sheet Lopinavir diamide **9**

**Fig. 12S:**
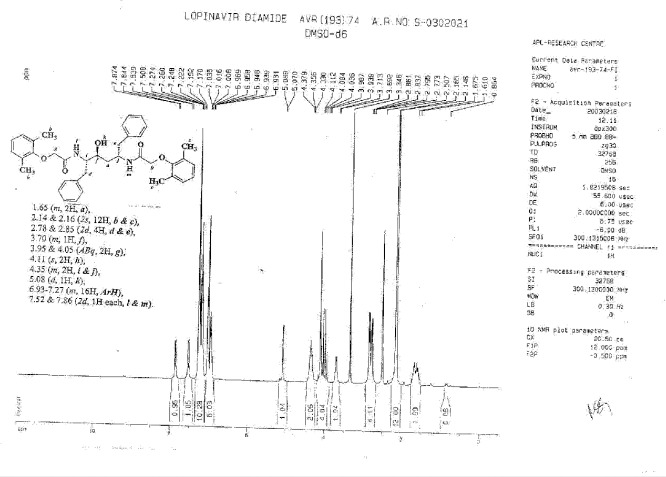
^1^H-NMR spectrum of Lopinavir diamide **9**

**Fig. 13S:**
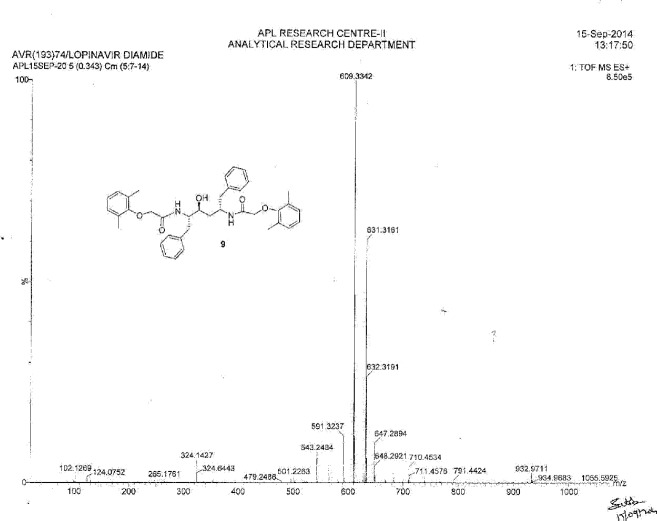
MS spectrum of Lopinavir diamide **9**

**Fig. 14S:**
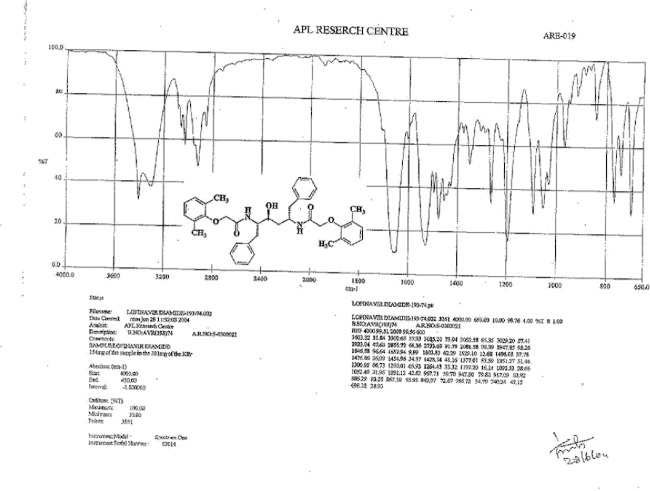
IR spectrum of Lopinavir diamide **9**

**Fig. 15S:**
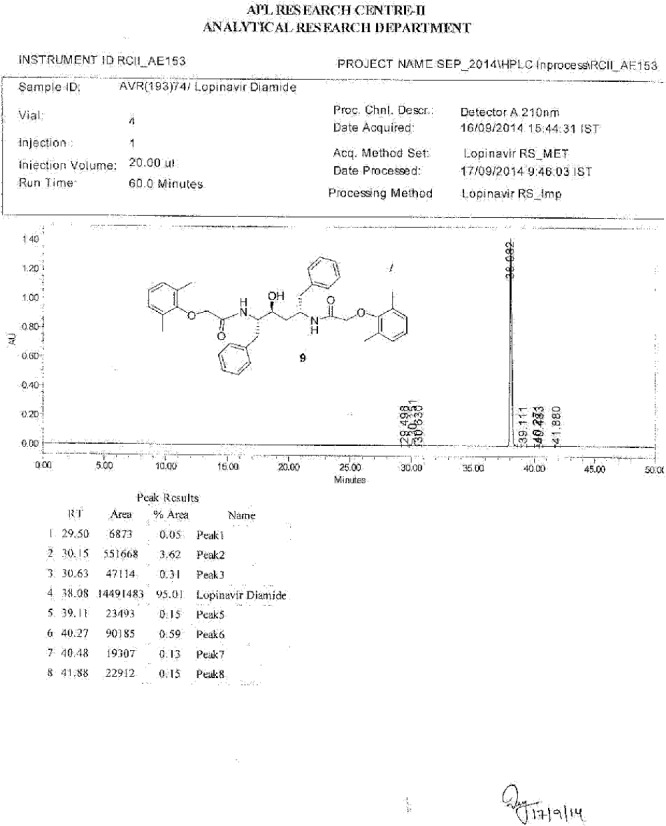
HPLC of Lopinavir diamide **9**

**Fig. 16S:**
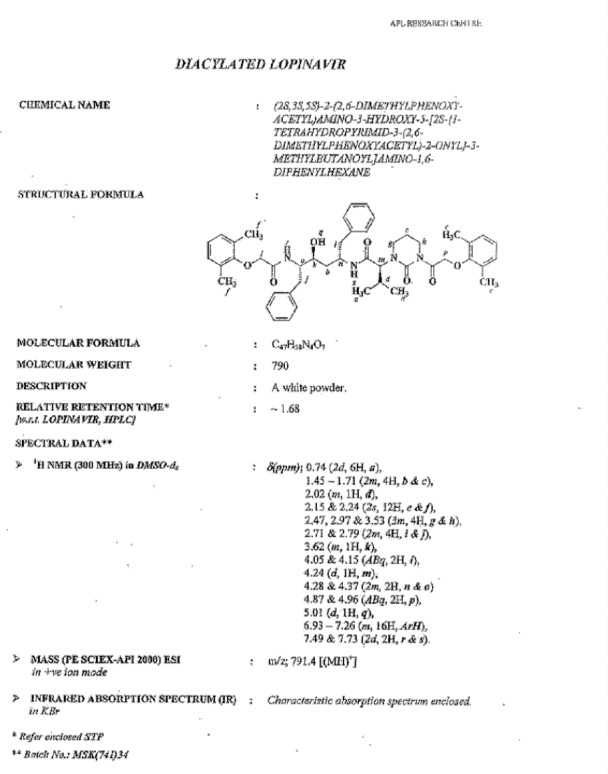
Data sheet of Diacylated Lopinavir **10**

**Fig. 17S:**
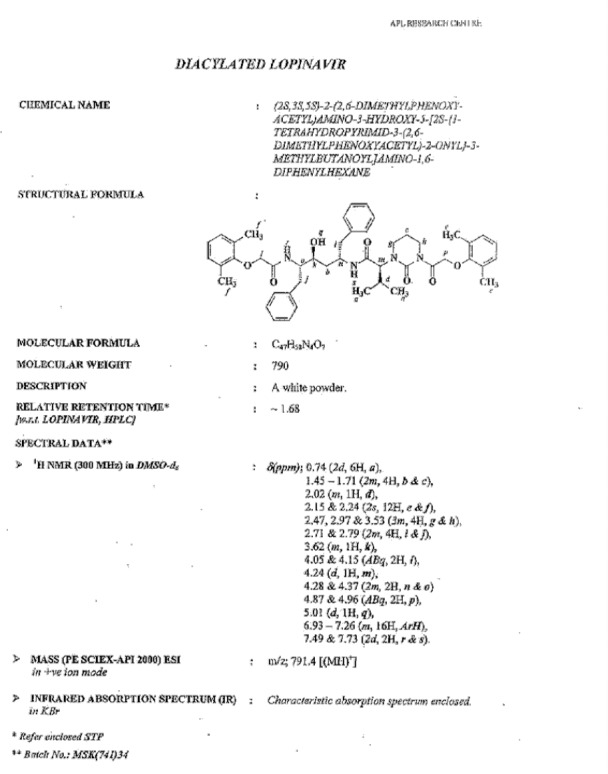
^1^H-NMR spectrum of Diacylated Lopinavir **10**

**Fig. 18S:**
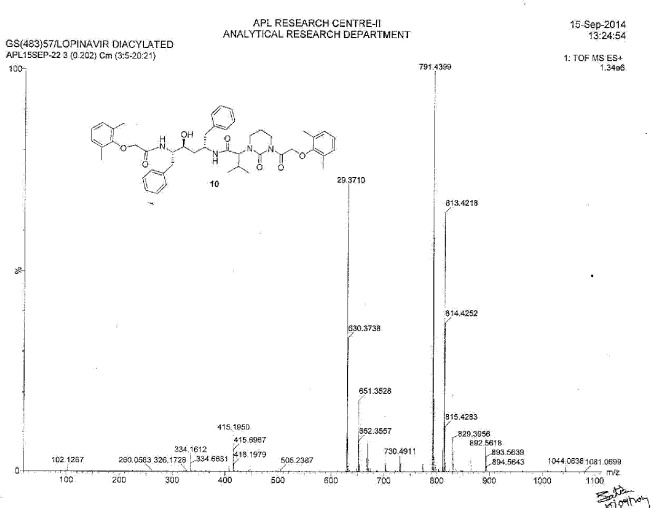
MS spectrum of Diacylated Lopinavir **10**

**Fig. 19S:**
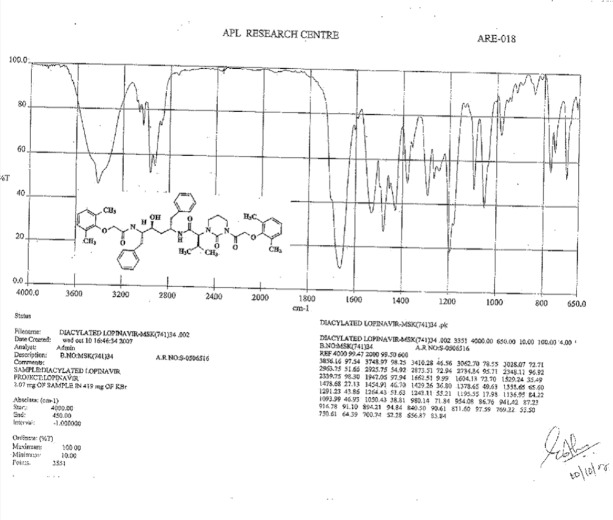
IR spectrum of Diacylated Lopinavir **10**

**Fig. 20S:**
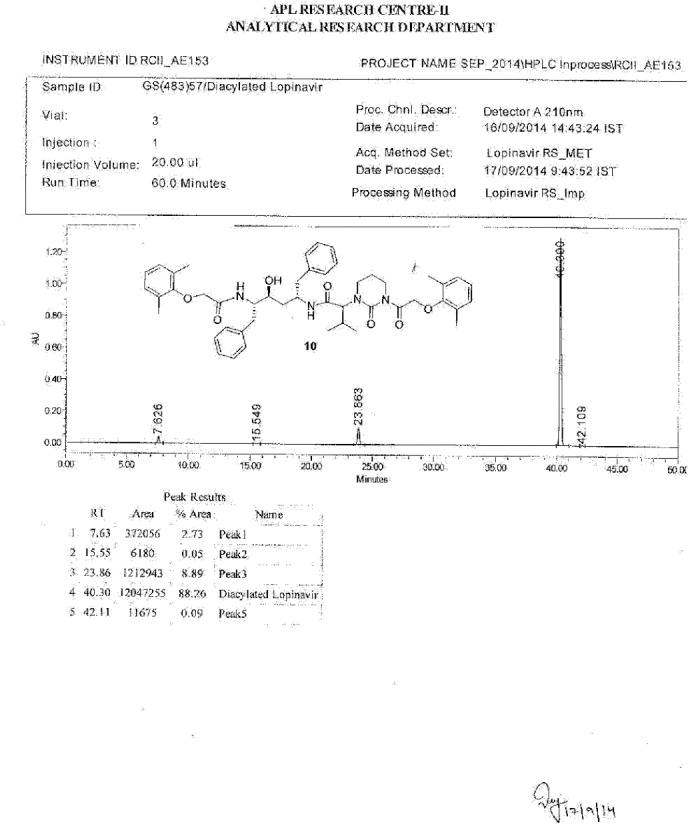
HPLC of Diacylated Lopinavir **10**

**Fig. 21S:**
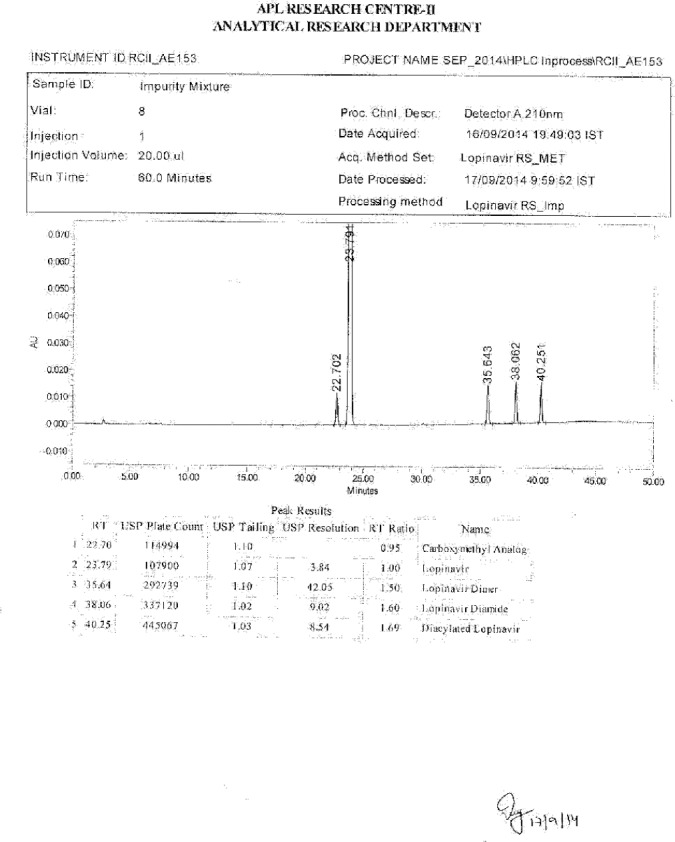
HPLC of Impurity mixture

## Supporting Information

The scanned ^1^H-NMR spectra of compounds **9–10**, the scanned ^13^C-NMR spectra of compounds **7** and **8**, the scanned MS spectra of compounds **7–10**, the scanned IR spectra of compounds **7–10**, the scanned HPLC of compounds **7–10** and the impurity mixture are available in the online version (Type: PDF, Seize: ca. 3.2 MB): http://dx.doi.org/10.3797/scipharm.1407-14.
